# ^1^H NMR based metabolic profiling distinguishes the differential impact of capture techniques on wild bighorn sheep

**DOI:** 10.1038/s41598-021-90931-y

**Published:** 2021-05-28

**Authors:** Galen O’Shea-Stone, Rachelle Lambert, Brian Tripet, James Berardinelli, Jennifer Thomson, Valerie Copié, Robert Garrott

**Affiliations:** 1 Department of Chemistry and Biochemistry, Montana State University, 103 Chemistry and Biochemistry Bldg., Bozeman, MT USA; 2 Department of Animal and Range Sciences, Montana State University, 103 Animal Biosciences Bldg., Bozeman, MT USA; 3 Department of Ecology, Montana State University, 310 Lewis Hall, Bozeman, MT USA

**Keywords:** Analytical biochemistry, Biological models, High-throughput screening, Metabolomics, Proteomic analysis, Ecology, Physiology, Systems biology, Zoology

## Abstract

Environmental metabolomics has the potential to facilitate the establishment of a new suite of tools for assessing the physiological status of important wildlife species. A first step in developing such tools is to evaluate the impacts of various capture techniques on metabolic profiles as capture is necessary to obtain the biological samples required for assays. This study employed ^1^H nuclear magnetic resonance (NMR)-based metabolite profiling of 562 blood serum samples from wild bighorn sheep to identify characteristic molecular serum makers of three capture techniques (dart, dropnet, and helicopter-based captures) to inform future sampling protocols for metabolomics studies, and to provide insights into the physiological impacts of capture. We found that different capture techniques induce distinct changes in amino acid serum profiles, the urea cycle, and glycolysis, and attribute the differences in metabolic patterns to differences in physical activity and stress caused by the different capture methods. These results suggest that when designing experiments involving the capture of wild animals, it may be prudent to employ a single capture technique to reduce confounding factors. Our results also supports administration of tranquilizers as soon as animals are restrained to mitigate short-term physiological and metabolic responses when using pursuit and physical restraint capture techniques.

Wild ruminants are important to human societies and have major impacts on the structure and function of ecosystems^1,2^. Globally, wild ruminants have experienced significant declines in numbers and distributions due to overharvest, anthropogenic alterations of landscapes, competition with domestic livestock, and exotic diseases introduced via comingling with livestock^3^. Prior to settlement of the temperate regions of western North America by Euro-Americans, a diverse suite of wild ruminants were found throughout the mountain and prairie environments, but by the early 1900s, populations were severely depleted or eliminated from much of the landscape^4^. The establishment of protective laws, development of wildlife science, and substantial investment of resources by society over the past century has restored populations of native wild ungulates to landscapes where suitable habitat exists. Substantial resources are invested into intensive management and research of restored wild ungulate populations to understand drivers of population dynamics and mitigate factors that limit demographic vigor. Despite intensive efforts by government agencies, however, restoration success for Rocky Mountain bighorn sheep (*Ovis canadensis*) has been modest. Historically bighorn sheep were broadly distributed throughout western North America with pre-settlement populations estimated at approximately 2 million animals^5^. Following a century of concerted restoration efforts, however, current bighorn sheep abundance is estimated at < 10% of historic levels^6^. Two major factors affecting the health and demographic vigor of bighorn sheep are nutritional limitations due to poor quality habitats, coupled with inter/intra-specific competition for forage^7^ and respiratory disease caused by bacterial pathogens^8^.

Wildlife scientists possess limited tools for assessing the nutritional health and disease status of bighorn sheep and other wild ruminants, inhibiting the understanding of wildlife-habitat relationships and the etiology of diseases. The rapid development of environmental metabolomics, i.e. the global analysis of small molecule metabolites present in organisms, cells, tissues, or biofluids, is expanding our abilities to investigate the interactions of organisms with their environment, and has the potential to facilitate the establishment of a new suite of tools for assessing the physiological status of important wildlife species, helping advance ecological understanding and enhance conservation^9^. A first step in developing metabolomics-based tools for assessing the health, nutritional, and physiological status of wild ruminants is to evaluate the impacts of various capture techniques on metabolic profiles as capture is a pre-requisite for obtaining the biological samples required for assays. Previous work has used a limited suite of serum biochemical and hematological assays to establish baseline values and to contrast the effects of various capture techniques, primarily to help inform wildlife managers on selection and execution of capture techniques that minimize serious physiological effects such as metabolic acidosis and capture myopathy, that can compromise the welfare of animals post-capture^10,11^.

The present study has explored the value of ^1^H nuclear magnetic resonance (NMR)-based metabolite profiling to identify characteristic molecular makers resulting from the three primary techniques (dart, dropnet, and helicopter-based captures) used to capture wild ruminants for research, conservation, and management. This work characterized the serum profiles of polar metabolites of wild bighorn sheep captured with different techniques to inform future sampling protocols for metabolomics studies of wild ruminants, and to provide insights into the physiological impacts of capture. We have found that in addition to stress indicators, different capture techniques induce very distinct and broad-spectrum serum metabolic changes in these wild animals.

## Methods

Capture and handling of animals reported herein complied with scientific guidelines and permits acquired from the State of Montana, the State of Wyoming, and the National Park Service. All animal capture and handling protocols were approved by Institutional Animal Care and Use Committees at Montana State University (Permit # 2011-17, 2014-32), Montana Department of Fish, Wildlife, and Parks (Permit # 2016-005), National Park Service (Permit # NPS 2014.A3), or Wyoming Game and Fish Department (Permit # 854).

### Study animals

A total of 562 serum samples were obtained from wild bighorn sheep in 14 populations distributed across Montana and Wyoming (Supplementary Table S1, Supplementary Fig. S1) that were captured, tagged, and sampled by wildlife management agencies as part of a regional ecological research program^10^. Animal captures occurred from December through March during the winters of 2014–15, 2015–16, and 2016–17, when all animals were on senescent native forages resulting in sub-maintenance diets. The majority of these animals, 385, were captured using net guns fired from a helicopter. This method required close pursuit by the helicopter, normally for 2–5 min, until a small net was deployed from a shoulder mounted gun that entangled the animal. A handler was place on the ground to physically restrain the captured animal via a blindfold and hobbles. Animals captured in remote wilderness were processed and released at the capture site, generally within 15–35 min of capture. In other situations with good ground access, the blindfolded and hobbled animals were placed in transport bags and slung under the helicopter on a long cable to a central processing site where they were processed, with blood samples normally collected between 20 and 60 min after capture. Large nets suspended over baited sites (dropnet) were dropped on 104 animals. Once the net was dropped, a large crew of handlers physically restrained the animals with blindfolds and hobbles, extracted each animal from the net, and carried it to a central processing area within 100 m of the dropnet site. Because the large nets captured 10–30 animals each, captured animals were queued for processing with blood samples generally drawn from 20 to 90 min following deployment of the nets. Ground-based delivery of immobilizing drugs, i.e. a cocktail of butorphanol, azaperone, and medetomidine; via dart rifles was used to capture 73 animals^12^. Animals were approached to within 5–15 m for effective dart delivery. Once stuck by the dart, the animals normally ran 5–20 m, and resumed pre-darting behaviors with their social group until the drugs began to take effect, causing the darted animal to bed down until sedated, normally 20–35 min following drug delivery. Sedated animals were blindfolded and hobbled, sampled, and drug antagonists administered, with processing time normally requiring 10–20 min.

For all capture techniques, a blood sample was drawn from the jugular vein of each animal and immediately placed under refrigeration until serum was harvested 2–6 h after capture. Serum was frozen at − 20 °C for transport to research facilities where all samples were stored at − 80 °C until further processed. The majority of the samples originated from unique animals, but small numbers of marked animals were repeatedly captured and sampled in consecutive years in three of the Wyoming herds.

### Sample preparation

Serum samples were prepared for small molecule polar metabolite extraction and ^1^H NMR metabolomics as follows: Samples were thawed at room temperature following storage at − 80 °C with reagents kept at − 20 °C until used. A 1:3 500 μL serum: 1500 μL acetone solution was added to 2 mL plastic, flat-cap conical vials^13^. The resulting solution was mixed thoroughly by inverting the sample tubes 10 times, and incubated at − 20 °C for 60 min to allow for protein precipitation, followed by sample centrifugation at 13,000×*g* for 30 min at room temperature. Clarified supernatants containing the polar metabolite mixtures were subsequently transferred to new 2.0 mL tubes and dried overnight using a Speedvac vacuum centrifuge with no heat, and stored at − 80 °C until further use. For NMR, dried metabolite extracts were resuspended in 600 μL of NMR buffer consisting of 25 mM of NaH_2_PO_4_/Na_2_HPO_4_, 0.4 mM of imidazole, 0.25 mM of 4,4-dimethyl-4-silapentane-1-sulfonic acid (DSS) in 90% H_2_O/10% D_2_O, pH 7.0.

### ^1^H NMR spectra acquisition and preprocessing

Samples in 2.0 mL centrifuge tubes were spun at 13,000 rpm for 2 min to remove any potential remaining debris, and 500 μL of each sample transferred into 5 mm Bruker NMR tubes. One dimensional (1D) ^1^H NMR spectra were recorded at 298 K (25 °C) using Montana State University’s Bruker 600 MHz (^1^H Larmor frequency) AVANCE III solution NMR spectrometer equipped with a SampleJet automatic sample loading system, a 5 mm triple resonance liquid-helium-cooled (^1^H, ^15^N, ^13^C) TCI cryoprobe, and the Topspin software (Bruker version 3.2). 1D ^1^H NMR experiments were performed using the Bruker ‘zgesgp’ pulse sequence with the following experimental parameters: 256 scans; a ^1^H spectral window of 9600 Hz; 32 K data points and a dwell time interval of 52 μs, amounting to an acquisition time of 1.7 s; and an additional 1 s relaxation recovery delay between acquisitions^14^.

For the verification of select metabolite identification (i.e. validation of metabolite ID), 2D ^1^H–^1^H total correlation spectroscopy (TOCSY) spectra were acquired for representative samples using the Bruker-supplied ‘mlevphpr.2/mlevgpph190 pulse sequences and the following experimental parameters: 256 (t_1_) and 2048 (t_2_) data points, 2 s (D_1_) relaxation delay, 32 transients per FID, ^1^H spectral window of 6602.11 Hz, and a 80 ms TOCSY spin lock mixing period. 2D ^1^H–^1^H TOCSY spectra were subsequently processed using the Topspin software (Bruker version 3.2).

### ^1^H NMR data analysis

**Figure 1:**
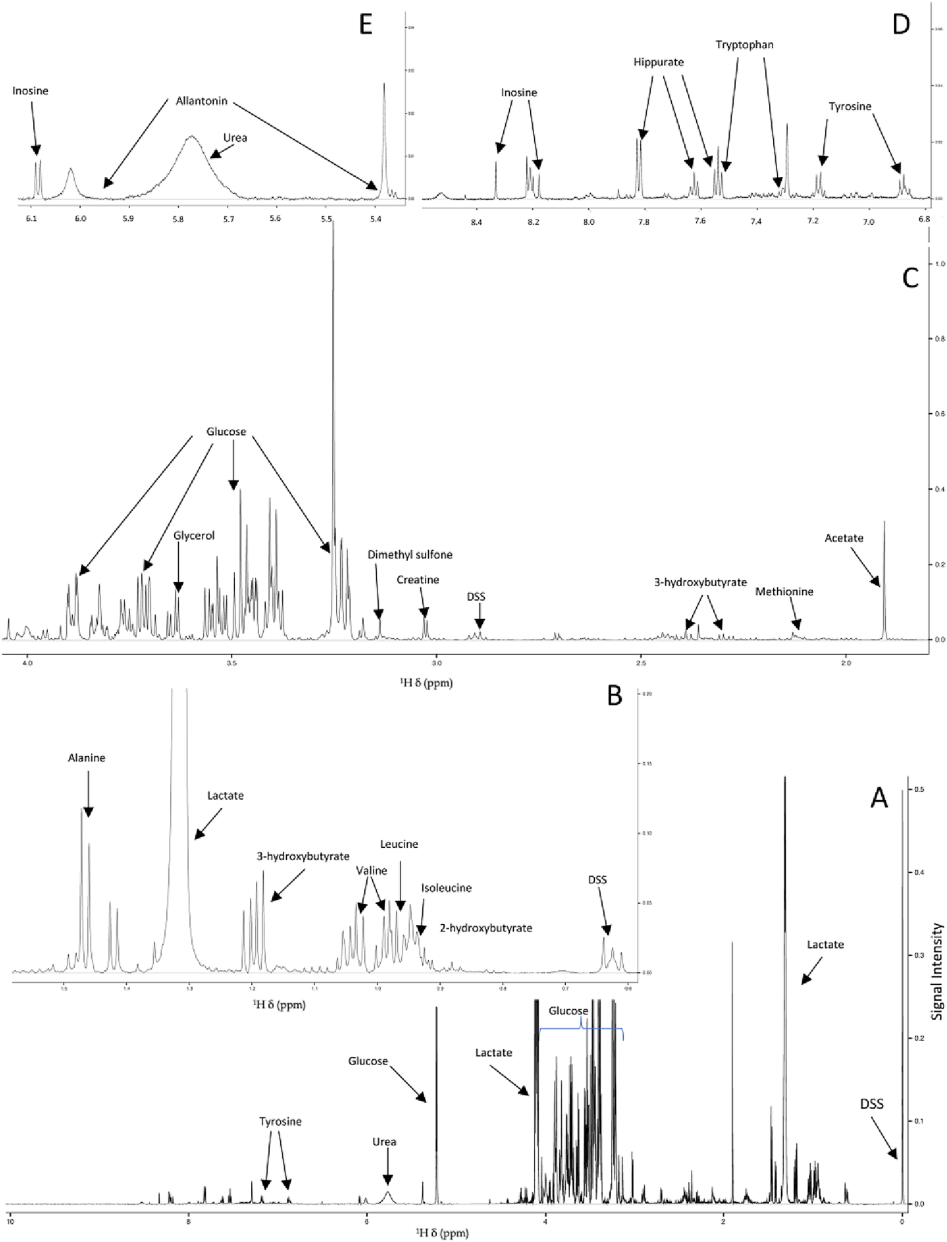
Representative 1D ^1^H NMR spectrum recorded on serum metabolite extracts of wild bighorn sheep on MSU’s 600 MHz NMR spectrometer. Panel (**A**) depicts the full ^1^H spectrum, spanning the ^1^H chemical shift region of ~ 0.0–10 ppm with select metabolites identified and quantified using Chenomx, and with several metabolite signals (lactate, glucose) off scale to render smaller signals visible. Panels (**B**–**E**) represent expanded regions of the full 1D ^1^H spectrum with (**B**) depicting the ~ 0.6–1.6 ppm; (**C**) ~ 1.9–4.1 ppm; (**D**) ~ 6.8–8.5 ppm; and (**E**) the ~ 5.3–6.1 ppm ^1^H chemical shift (δ, ppm) spectral regions, with signals from select metabolites labeled. DSS denotes the reference compound, sodium trimethylsilylpropanesulfonate.

Spectral analysis, processing, and metabolite annotations were performed using the Chenomx NMR Software (Version 8.4; Chenomx Inc., Edmonton, Alberta, Canada), following Chenomx protocols and published NMR metabolomics data analysis approaches^15–17^. Spectral baselines were adjusted using the Chenomx spline automatic adjustment (Whittaker function), followed by inserting manually baseline breakpoints to achieve flat and well-defined spectral baselines. Line broadening, phase correction, and shim correction were employed following Chenomx protocol recommendations and previously reported data processing approaches^18,19^. ^1^H Chemical shifts were referenced to DSS whose NMR signal was set at 0.0 ppm, and the NMR signal from imidazole was used to correct for small chemical shift changes due to slight pH variations. NMR signals were quantified from relative signal intensity with DSS as reference, normalized to sample volumes, and annotated by matching chemical shift and spectral splitting patterns to those of reference spectra accessible through the Chenomx 600 MHz (^1^H Larmor frequency) spectral database of small molecule metabolites^15,16^ (Fig. 1). Using the Chenomx software, complex NMR spectral patterns obtained from the 1D ^1^H NMR spectra of resulting metabolite mixtures were deconvoluted and used for identification and quantification of 49 distinct metabolites from the three different animal capture techniques. A representative annotated NMR spectrum is shown in Fig. 1. Tables of the concentrations (in μM) of 49 unambiguously identified metabolites were then exported from the Chenomx software for multivariate and univariate statistical analysis.

### Statistical methods

Pre-processing parameters for statistical analysis were implemented using Metaboanalyst 4.0, and included replacing missing values for metabolite concentrations that were not observed in some samples but observed in others with 1/5 of the minimum positive value to their corresponding variables^20^. This step was applied to 0.2% of the samples and no discernable pattern of missing values was observed within any specific capture group. Logarithmic transformation (log base 2) was applied to the data to adjust for potential skewing of data distributions^21^. Normalization procedures are necessary prior to statistical analysis of metabolomics data to reduce systematic variations, and to correct for changes originating from intrinsic biological sample variations within groups^22^. To this effect, sample-wise normalization to a constant sum (centering) was applied, as well as auto-scaling (i.e. centering each variable around the mean and dividing by the standard deviation)^23^. Of the 562 samples originally included in this analysis, a subset of suspected extreme observations was observed (n = 21). The metabolite concentration data corresponding to these samples were examined and found to be missing metabolite information from 18% of the time points collected, which led us to omit these 21 samples from further analysis.

**Figure 2:**
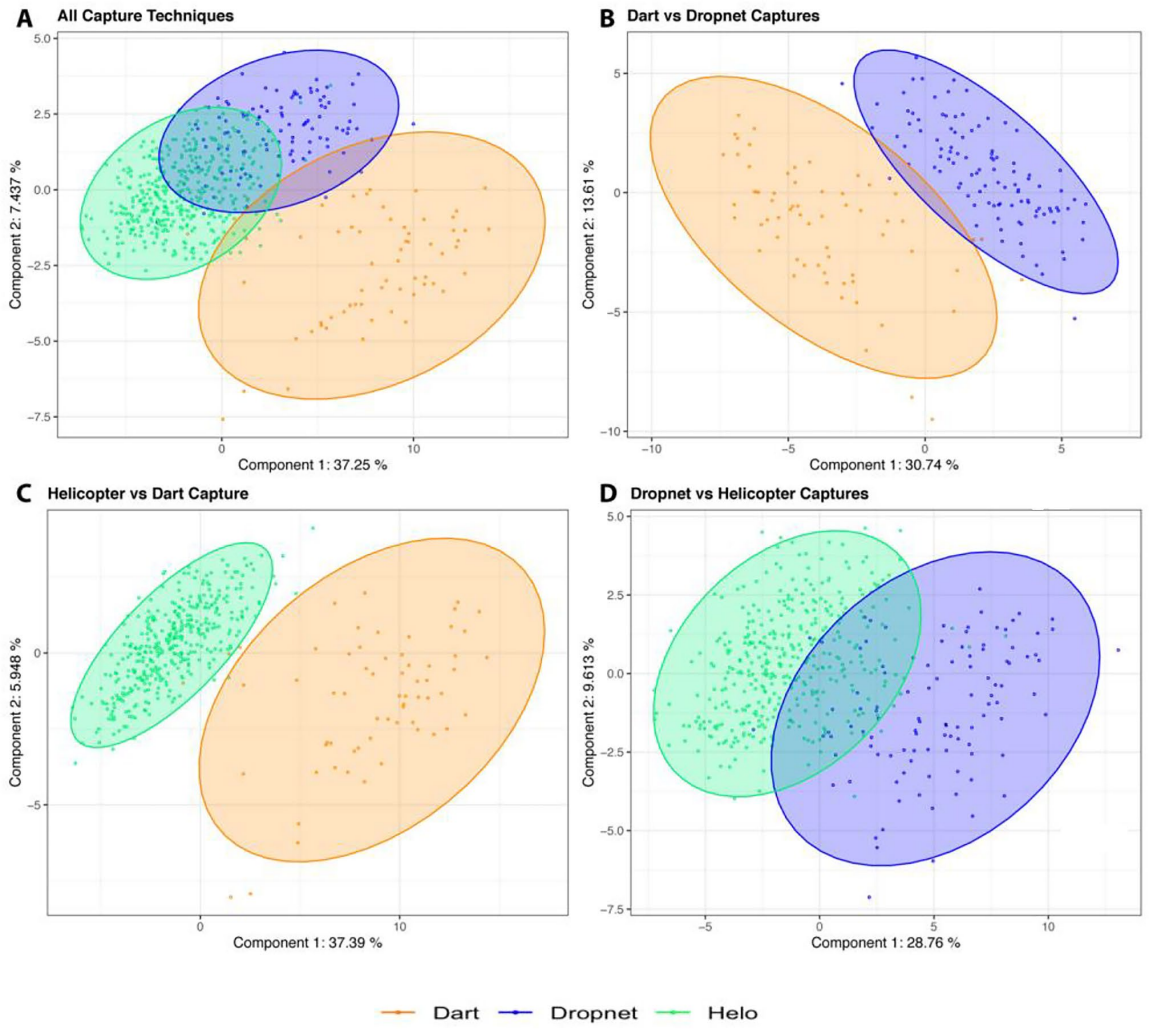
Two-dimensional partial least squares discriminant (2D-PLS-DA) scores plots generated from polar metabolite profiles including (**A**) all capture methods including dart (orange), dropnet (blue), and helicopter (helo, green) methods; (**B**) dart versus dropnet; (**C**) dart versus helicopeter capture methods; and (**D**) helicopter and dropnet captures methods. The number of animals in each group consisted of n = 73 for dart; n = 93 for dropnet; and n = 375 for helicopter capture, with shaded ellipses representing 95% confidence intervals. PLS-DA validation metrics included (i) Q^2^ = 0.80 (component 5), R^2^ = 0.82 (component 5), classification error rate (CER) < 0.10 (component 5), Area under ROC Curve (AUC) > 0.95 (Dart vs. others and Helo vs. others) (component 1), (ii) Q^2^ = 0.85 (component 5), R^2^ = 0.87 (component 5), CER < 0.03 (component 5), AUC = 0.95 (component 1), (iii) Q^2^ = 0.86 (component 5), R^2^ = 0.89 (component 5), CER < 0.02 (component 5), AUC = 0.99 (component 1), and (iv) Q^2^ = 0.62 (component 5), R^2^ = 0.69 (component 5), CER < 0.04 (component 4), AUC > 0.96 (component 1). Permutation tests (n = 2000) for all PLS-DA models p < 0.001.

Initial multivariate statistical analysis was performed using Metaboanalyst 4.0 and included 2D principal component analysis (2D-PCA) and hierarchical clustering analysis (HCA)^20^. HCA was conducted using a Euclidean distance measure and Ward clustering algorithm. All 49 metabolites identified and quantified in almost all samples were used to assess whether differences in serum polar metabolite patterns (i.e. differences in metabolite levels) could discriminate and separate the different animal capture groups. To further discriminate between groups, Partial Least Squares Discriminant Analysis (PLS-DA) was performed, as this method is widely used to assess the maximum covariance between a dataset and class labels. Our team used R, along with the caret package, MixOmics and MetaboanalystR packages for PLS-DA modeling, PCA analysis, one-way ANOVA (Tukey’s post-hoc test), and volcano plot analysis^20,24–26^. A potential issue with PLS-DA is that the approach can be susceptible to model overfitting, leading to separations by class that may not be real or are exaggerated^27^. To assess model validation, the diagnostic ability of the PLS-DA classifier system was assessed using classification error rate (CER) analysis, area under the receiver operating characteristic curve (AUROC), permutation tests (n = 2000), and evaluating Q^2^ and R^2^ values associated with the PLS-DA models (Fig. 2, Supplementary Fig. S2)^28^. Validity of the PCA analysis was assessed using similar parameters, using the caret and MixOmics software packages in R^25,26^.

## Results

Two-dimensional principal component analysis (2D PCA) using Metaboanalyst and R programs was employed to evaluate whether polar serum metabolite level differences could separate animals from the different capture groups, and provided encouraging information about separate group clusters, leading to supervised multivariate statistical approaches to further analyze the significance of the metabolite profiling data.

Partial least squares discriminant analysis (PLS-DA) scores plots enabled the visualization of metabolic profile differences within groups of animals that were captured by the three different capture techniques (Fig. 2A), as well as between groups (Fig. 2B–D). PLS-DA modeling of the metabolomics data for all capture techniques, as well as for dart versus helicopter, dart versus dropnet, and dropnet versus helicopter successfully met model validation metrics including ROC curve profiles, Q^2^ parameter, classification error rates and permutation tests (Fig. 2, Supplementary Fig. S2A–D).

**Figure 3:**
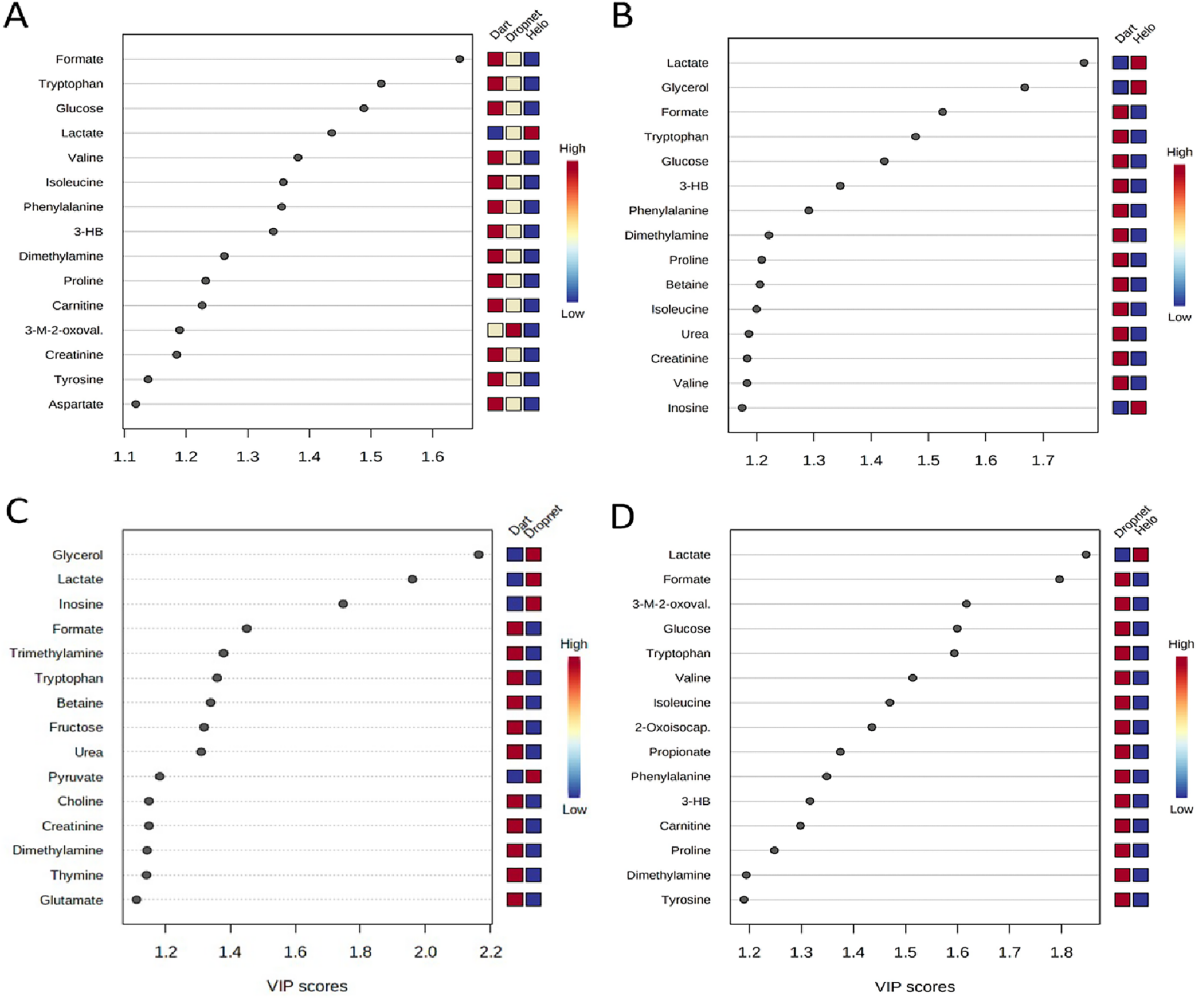
Variable of Importance (VIP) scores plots depicting the 15 most significant metabolites (VIP > 1.2) contributing to the animal group separations observed in the PLS-DA model analyses done using MetaboAnalyst. VIP scores plots are shown for (**A**) all three capture groups; (**B**) helicopter vs. dart capture groups; (**C**) dart vs. dropnet capture groups; and (**D**) and helicopter vs dropnet capture groups. Abbreviations denote: *3-HB* 3-Hydroxybutyrate, *3-M-2-*oxoval. 3-Methyl-2-oxovalerate, *2-*Oxoisocap. 2-Oxoisocaproate. Blue and red boxes denotes metabolite levels that are lower or higher, respectively, between animal groups, with pale yellow indicating intermediary levels.

2D-PLS-DA scores plots (Fig. 2) demonstrated that the different animal capture techniques (dart, dropnet, and helicopter) lead to significant changes in the serum metabolite profiles of the captured animals. When comparing the three capture techniques together, the metabolites level differences associated with top scores in PLS-DA variable importance in projection (VIP > 1.2) plots included: formate, tryptophan, glucose, valine, glycerol, phenylalanine, 3-hydroxybutyrate, dimethylamine, proline, and carnitine which were found in lower concentrations in the serum samples of animals captured by helicopter compared to those of animals captured by dart, and intermediate levels for animals captured by dropnet (Fig. 3). This is in contrast to the concentrations of lactate, which was lower in animals captured via dart (Fig. 3A).

**Figure 4:**
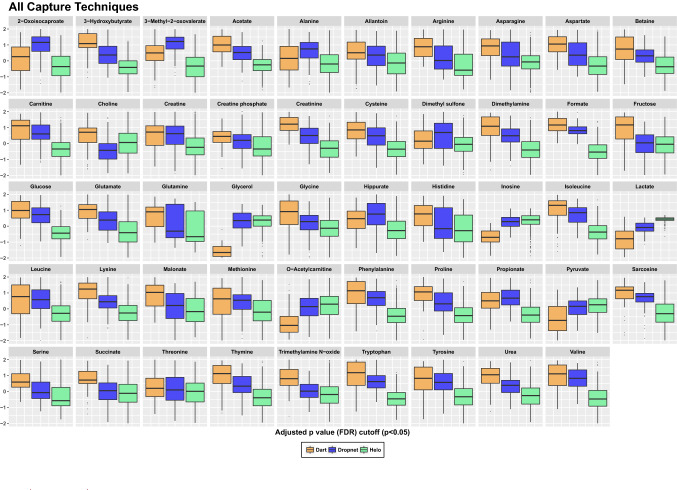
One-way parametric ANOVA analysis of metabolite levels revealing 49 significant metabolites (p < 0.05), when concentration differences reported as normalized values are examined between three capture techniques. Analysis was performed using Tukey’s HSD post-hoc analysis. Whiskers indicate ± 1.5* interquartile range (IQR) observations and values > 1.5 and < 3 *IQR are represented as black dots. Dart captures are shown in orange, dropnet captures in blue and helicopter capture in green.

**Figure 5:**
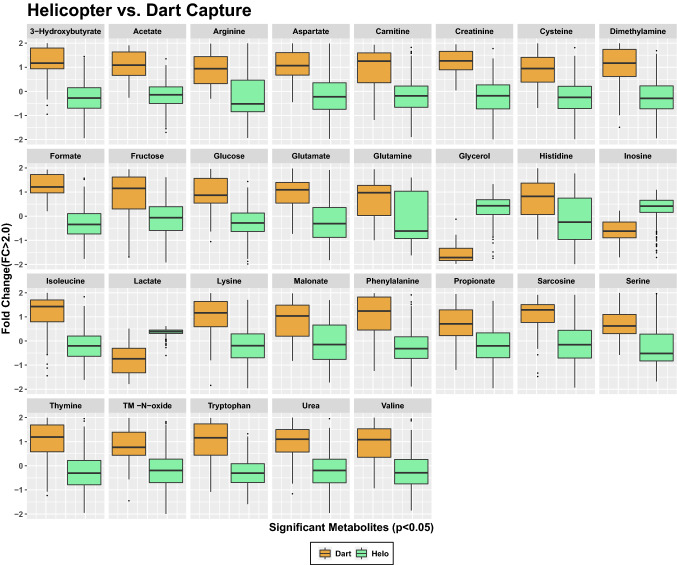
One-way parametric ANOVA analysis revealing statistically significant (FC > 2.0; p < 0.05) metabolites that differentiate wild bighorn sheep captured by dart (orange) versus helicopter (green). Whiskers indicate ± 1.5* IQR observations and values > 1.5 and < 3 *IQR are represented as black dots.

ANOVA analysis assessed differences in fold change (FC) and identified statistically significant metabolites that differentiate samples obtained from the three different capture techniques (Fig. 4). Metabolites that met significance threshold of p < 0.05 were identified (Fig. 4) and included all 49 polar metabolites identified by NMR. Of these, the most significant differences included lower concentrations of lactate, inosine, and glycerol in the dart-captured animals, with comparable levels measured in serum samples of animals captured either via the dropnet or helicopter methods. When examining capture techniques pairwise, the largest number of significant metabolites (p < 0.05; FC > 2.0) separating capture groups were observed in the analysis of the helicopter and dart captured animal groups (Fig. 5).

Additional PLS-DA analyses were undertaken to assess the extent of serum metabolite changes in pairwise comparisons of capture techniques. The first set included dart versus helicopter, as these two capture techniques yielded the most distinct serum metabolomes (Fig. 2C). This analysis revealed higher levels of formate, tryptophan, glucose, 3-hydroxybutyrate, phenylalanine, dimethylamine, and proline in the serum samples of animals captured by the dart method compared to those captured by helicopter (Supplementary Table S3, Fig. 3B), similar to what was observed when the metabolites profiles obtained from all three techniques were analyzed together. The pairwise analysis of dart versus helicopter groups also revealed lower concentrations of lactate, glycerol, and inosine in the serum samples of dart-captured animals (Figs. 3B, 5, Supplementary Table S2). To visualize the contribution of specific metabolite differences to the separation observed in the PLS-DA model of the different capture groups, loading vectors were plotted as horizontal plots indicating the highest/lowest mean value for each metabolite. The analysis of PLS-DA loading vectors importance values highlighted choline, glycerol, 2-oxoisocaporate, 3-methyl-2-oxovalerate, lactate, inosine, and alanine as significant metabolites driving the separation between these two capture groups (threshold <  ± 0.25, Supplementary Fig. S3).

PLS-DA analysis of serum metabolite profiles collected from bighorn sheep animals captured via dart versus dropnet methods (Fig. 2B) revealed a clear separation between the two groups. In particular, three metabolites were identified by VIP scores as being most significant and included: glycerol, lactate, and inosine, which were all lower in concentrations in the serum samples of dart-captured animals compared to dropnet (Fig. 3C). Lower, albeit less pronounced changes in 3-methyl-2-oxovalerate, 2-oxoisocaproate, and pyruvate were observed, being lower in dart-captured animals (Fig. 4). Other metabolites that were identified as significant discriminators between the dart versus dropnet capture groups (VIP > 1.2) included formate, trimethylamine, tryptophan, betaine, fructose, urea, all being in lower concentrations in the serum samples of dropnet-captured animals compared to the dart-captured group (Fig. 3C). Subsequent univariate analysis identified significant metabolites with fold change (FC) > 1.5 and p < 0.05, and included lower levels of creatine phosphate and glutamate in the dropnet group, and lower levels of glycerol, inosine, lactate, and *O*-acetylcarnitine in the dart-captured group (Supplementary Fig. S4).

Lastly, PLS-DA modeling of the serum metabolome profiles of helicopter vs dropnet captured animals (Fig. 2D) revealed that the two groups were slightly distinct from each other, at least based on fewer polar serum metabolite profile differences identified, suggesting that dropnet and helicopter capture methods may be inducing comparable metabolic responses in the animals (Fig. 2D). These observations are further supported by univariate statistical analysis, which revealed that metabolite level changes in only three metabolites, 3-methyl-2-oxovalerate, formate, and propionate were significant and exhibited FC > 2.0 and p < 0.01 when comparing the serum metabolite profiles of animals captured by dropnet versus helicopter, with all being higher for the dropnet group (Supplementary Fig. S5). A complete list of all metabolites identified and quantified, with corresponding means and standard deviations, is reported in Supplementary Table S2.

## Discussion

Examining the serum metabolome profiles of bighorn sheep captured by the three primary techniques used to capture wild ungulates revealed significant changes in polar metabolite levels between the different animal groups, and trends that persisted throughout the analyses when directly comparing, in a pairwise fashion, specific capture techniques. Results from PLS-DA modeling and analysis of the top 15 metabolites that contribute most (VIP > 1.2) to the separation of the three capture groups revealed that amino acid levels of tryptophan, valine, isoleucine, phenylalanine, and proline were highest in animals captured by dart, with intermediate levels in animals capture using dropnets, and lowest in animals captured using the helicopter method (Fig. 3A). One-way ANOVA analyses identified additional amino acids that displayed similar decreasing level trends from dart to dropnet to helicopter capture (dart > drop net > helicopter) methods, and included arginine, asparagine, aspartate, cysteine, glutamate, and glutamine, glycine, histidine, leucine, lysine, serine, and tyrosine (Fig. 4). These metabolite level changes suggest a shift in amino acid metabolism, and a potentially higher catabolism of these compounds as a function of increasingly more energetically intense and possibly more stressful capture methods such as helicopter capture.

Of these amino acids, aspartate, glycine, and glutamate function as precursors for neurotransmitter synthesis, and may therefore be valuable indicators of the capture techniques’ impacts on animal health and changes to their physiological state. Glutamate is a fundamental component of nitrogen excretion in the urea cycle, and its lower serum levels in animals captured by helicopter support the idea of altered metabolite flow through the urea cycle. In addition to these patterns, decreasing levels of aspartate were observed in samples of dropnet and helicopter captured animals compared to the levels found in the dart-captured animals. The change regarding urea cycle alterations also manifested itself in differential serum urea levels, with fold changes (FC) between the groups decreasing significantly with capture techniques, with a mean FC difference of 1.4 for the dart-captured group, 0.26 for the dropnet-captured group, and − 0.3 for the helicopter-captured animals (Supplementary Table S2). As urea recycling is a prominent feature of ruminant metabolism and urea flux can rapidly change, the urea concentration changes observed between the three capture techniques support an impact on urea cycle intermediates^29^. While the trend of an overall decrease in urea cycle intermediates parallels a similar trend in amino acid concentrations, the extent to which amino acid metabolism is linked to changes in urea cycle activity is difficult to evaluate due to the nature of nitrogen recycling in the rumen of these ruminants.

Other metabolites found in significantly higher concentrations in the serum samples of dart-captured animals compared to the two other techniques included: formate, glucose, 3-hydroxybutyrate, dimethylamine, carnitine (Fig. 3A). Propionate, which was observed to be higher in the dart and dropnet captured animals than that of helicopter captured animals (Fig. 4) is of interest, as it is the main precursor for glucose synthesis in the liver of ruminants^30^, and potentially reflect a higher dependence of ruminants on gluconeogenesis due to the almost complete conversion of available dietary carbohydrates to volatile fatty acids in the rumen^31^. As animal capture via nets increases physical activity as the animals struggle to free themselves from entanglement, generally resulting in longer times animals are under physical restraint, as well as the increased physical exertion and stress as they attempt to flee the pursuing helicopter, the observed decrease in serum propionate levels may reflect increased needs to generate glucose de novo via gluconeogenesis.

This interpretation of the metabolite data is reinforced by the observation of significantly elevated levels of *O*-acetylcarnitine in the drop net and helicopter net gun animal capture groups compared to the darted animals (Fig. 4). As an important element of the carnitine/acyl-carnitine shuttle and import of fatty acids into the mitochondria for β-oxidation, acyl-carnitine is a major contributor to the flow of acyl groups into the TCA cycle, and a robust indicator of cardiac output and, by extension, TCA cycle activity levels in mammals^32^. Additional metabolites that displayed distinctly increasing trends based on capture method (dart < dropnet < helicopter), including glycerol, inosine, lactate and pyruvate (Fig. 4). Of these, pyruvate and lactate are particularly relevant to capture techniques, as they represent major components of anaerobic glycolysis. Greater levels of these metabolites in the serum profiles of dropnet and helicopter-captured animals may reflect the greater physical exertion experienced by dropnet and helicopter-captured animals compared to dart-captured animals and the generally longer times animals are under physical restraint. These differences in pyruvate and lactate levels are consistent with our observations that serum glucose levels are lowest in animals captured by helicopter, higher in the dropnet group, and highest in dart-captured animals (Fig. 3A). While we acknowledge that the drugs employed for chemical immobilization have the potential to influence metabolic profiles, we could not discern any notable differences in the metabolomics profiles of darted animals that we could attribute to the drugs. As described, most of the influential metabolites that discriminate the three capture techniques are primarily associated with physical exertion and stress.

Analysis of the serum profiles of animals captured using the immobilizing dart method compared to those of animals captured using helicopter net gun capture, revealed persistence of several of the metabolite level trends that were observed when evaluating metabolome differences between all three techniques (Figs. 4, 5). PLS-DA analysis indicated significantly elevated levels of glycerol, lactate, and inosine in the helicopter capture group compared to the dart capture group (Fig. 3C). Lower levels of inosine in darted animals paralleled trends in elevated lactate levels for the helicopter capture group, potentially representing a robust indicator of the metabolic impact of the two different capture techniques, as the serum concentration of inosine was almost 8 times greater in the helicopter capture group compared to dart (Fig. 5, Supplementary Table S1). A similar trend was noticeable for glycerol, as its serum levels were over two orders of magnitude higher in helicopter versus dart-captured animals (Supplementary Table S2). These metabolite level changes suggest an increase in fatty acid catabolism in the helicopter captured animals, due to increased energy (ATP) needs resulting from the increased exertion as these animals attempt to evade capture. Changes in the levels of these three metabolites (glycerol, lactate, inosine) highlight the impact of the dart versus helicopter capture techniques on the serum metabolite profiles of animals captured by these two very different approaches.

The pairwise analysis of the polar metabolite profiles of dart versus helicopter-captured groups also highlighted specific changes in the serum concentrations of amino acids, including tryptophan, lysine, and cysteine, which serves as a source of precursors for TCA cycle activity, via production of pyruvate, which was increased in the serum profiles of animals captured by helicopter (Fig. 5). In contrast, serum levels of asparagine, aspartate, valine, and proline were significantly lower in the helicopter-captured animals (Fig. 5). These amino acids are vitally important for diverse central carbon energy metabolic processes, and are used to generate additional intermediates such as fumarate, succinyl-CoA, and α-ketoglutarate. Changes in these amino acid levels may thus reflect significant changes in central carbon metabolism and energy-generating processes in dart versus helicopter captured animals, and the significant impact of these capture techniques on the physiology of wild bighorn sheep. Other metabolites found in lower concentration in the serum samples of helicopter-captured animals included formate, dimethylamine, and urea (Fig. 5). Changes in the levels of these metabolites reflected changes in urea metabolism which mimicked what was observed when comparing all capture techniques (Figs. 4, 5), and provide additional evidence for the impact of capture technique on nitrogen metabolism and the urea cycle.

In animals captured using the dropnet method compared to the immobilizing dart technique, similar serum metabolite patterns to those identified in animals captured by helicopter versus dart were observed. PLS-DA analysis indicated lower concentrations of serum glycerol, lactate, and inosine in the dart versus dropnet-captured animals (Fig. 3B), very similar to what was observed when comparing the dart versus helicopter groups (Supplementary Fig. S4). Interestingly, ketoleucine (2-oxoisocaproate) levels were higher in the sera of animals captured by dropnet compared to helicopter in which it was the lowest (Fig. 4). This apparent change in ketoleucine concentrations suggests that capture may induce a change in branch chain amino catabolism which is further supported by observations of decreasing levels of valine, leucine and isoleucine from dart to dropnet to helicopter capture techniques (Fig. 4)^33^. Similar to what was observed when comparing the serum profiles of dart versus helicopter-captured animals, the levels of several key amino acids involved in energy production were altered, all lower in the serum samples of dropnet-captured animals compared to dart-captured animals, and included lysine, arginine, cysteine, glutamate, phenylalanine, serine, and tryptophan (Supplementary Fig. S4). The extent of these changes were within the same orders of magnitude as to what was observed in the dart versus helicopter capture. These metabolite patterns suggest comparable shifts in central carbon energy metabolism when animals were captured by the dart compared to the helicopter or dropnet method, the latter yielding very similar metabolite profiles to those observed for animals captured by helicopter, albeit in a seemingly less dramatic fashion as reflected in fewer specific metabolite level changes being significant (18 vs 29 when comparing dropnet and helicopter to the dart-captured group, respectively). A key metabolite discriminating dart from dropnet capture techniques involved choline, which was significantly lower in concentration in the dropnet-captured animals (FC = − 0.4) compared to the dart-captured group (FC = 0.6) (Fig. 4 and Supplementary Table S2). This trend in choline level was also noticeable when all three capture techniques were analyzed together, with dropnet and helicopter serum samples exhibiting lower choline levels compared to dart, similar to what was seen when the dart and helicopter groups were compared (Fig. 4 and Supplementary Table S2). The importance of choline level changes was also highlighted in the PLS-DA loading vectors importance values (Supplementary Fig. S3), with choline being one of the driving factors that separated the three capture groups, specifically contributing to the separation of the dropnet group from the two other capture groups (Supplementary Fig. S3). These data suggest that dropnet and helicopter capture techniques have a greater impact on key metabolic pathways associated with choline metabolism including the Kennedy pathway, which accounts for ~ 95% of choline utilization to generate phosphatidylcholine and phosphatidylethanolamine^34^. Other potentially impacted processes included intermediates of the one-carbon metabolism cycle, of which choline and betaine are main contributors^35^. Additional evidence supporting changes in one-carbon cycle involved the decrease in betaine levels when all three capture techniques were compared, with dart having highest level of betaine, followed by dropnet, and then helicopter (Fig. 4). Overall, the trends in metabolite level changes observed when comparing dart versus helicopter capture groups persisted in the dart versus dropnet-capture comparisons, with a few exceptions as presented above.

The polar metabolite profiles obtained from serum samples of animals captured by dropnet and helicopter were more similar to each other than those of dart-captured animals. Nevertheless, PLS-DA analysis indicated 15 metabolites that contributed to the separation of dropnet versus helicopter capture groups (Fig. 2D), which were all lower in concentration in the serum samples of helicopter-captured animals except for lactate (Fig. 3D). Metabolites whose levels were higher in the dropnet capture group compared to the helicopter group included formate, 3-methyl-2-oxovalerate, glucose, tryptophan, valine, isoleucine, 2-oxoisocaproate, proprionate, phenylalanine 3-hydroxybutyrate, carnitine (Fig. 3D, VIP > 1.3). These changes were consistent with the trends observed when the serum profiles of dart-captured animals were compared to those of the helicopter and the dropnet-captured groups (Fig. 4). Some serum amino acid levels were significantly lower in the helicopter capture group compared to dropnet, but the magnitude of these differences was less pronounced than the one observed when the dart-capture group was compared to the helicopter or to the dropnet capture groups. The differentiating trends between helicopter and dropnet were reflected in the PLS-DA models, although the validation metrics (Q^2^ ~ 0.6, R^2^ ~ 0.7 and CER < 0.08) were marginal, suggesting that the PLS-DA model may be slightly overfit for this pairwise capture group comparison (Supplementary Fig. S2). Univariate and volcano plot analysis indicated that level changes of only three metabolites, including formate, propionate, and 3-methyl-2-oxovalerate contributed to the separation of the dropnet from the helicopter capture groups (Supplementary Fig. S5).

In conclusion, we have found that different animal capture techniques result in distinct and broad serum metabolic changes in wild bighorn sheep. Serum metabolite profile differences were most significant when the dart-captured animals were compared to the other animal groups captured by dropnet or helicopter methods. Metabolite level changes were less pronounced when the serum metabolite profiles of dropnet-captured animals were compared to those of the helicopter-captured group.

The differences in metabolic profiles documented in this study were attributed primarily to differences in physical activity and stress caused by the different capture methods. Both dropnet and helicopter capture rely on nets with consequential physical struggles as the animals attempt to escape entanglement. Both also involve significant time between capture and animal restraint with blindfolds and hobbles aimed at reducing physical activity, but no doubt causing stress, continued muscle exertion, and elevated heart, respiration, and metabolic rates. While there existed considerably variability in the time animals were manipulated when subjected to each capture technique, in general, animals captured with dropnets and helicopters were physically retrained for long periods of time. In contrast, cautiously approaching animals on the ground and delivering immobilization drugs via a dart rifle appear to result in minimal physical exertion and, while darted animals were also blindfolded and hobbled for all handling, processing and sampling, physical exertion and stress appear to be minimal due to sedation. This study has thus demonstrated that the three animal capture techniques examined here, which are the primary techniques employed to capture most wild ruminants, have wide ranging impacts on the metabolism of bighorn sheep, as reflected in significant and broad ranging changes in serum polar metabolite profiles. Most notable appears to be a significant shift in central carbon energy metabolism due to the nature of the type of capture technique employed.

The field of metabolomics has considerable potential to enhance the assessment of the health and physiological state of wild animals, and to guide efforts aimed at improving their conservation and management. Of particular interest for wild ruminants is the development of quantitative analytical tools to accurately characterize their body reserves, nutritional status, and disease state, which are the primary limiting factors influencing wild animal populations.

Controlled experimental studies with captive animals will provide the most rigorous approach to developing metabolomics-based tools, but ethical constraints limit experimental protocols involving disease processes, and preclude experimental protocols that mimic the type of severe and prolonged nutritional deprivation routinely experienced by wild ruminants inhabiting seasonal environments. Thus, complementary observational studies of wild animals will be needed to realize the full potential of metabolomics for wildlife conservation and management. Our findings suggest that when designing such studies that require the capture of wild animals, it may be prudent to employ a single capture technique, if possible, to reduce confounding factors that may alter serum metabolome profiles. The more dramatic changes that were observed in the polar serum metabolite profiles of animals captured using the dropnet and helicopter techniques suggest that administration of tranquilizers as soon as animals are restrained may be warranted to mitigate short-term physiological impacts^36,37^.

## Supplementary Information

Supplementary Information.

## Data Availability

NMR spectra and raw data will be deposited in the MetaboLights data repository for public access following acceptance of the manuscript for publication. The data will also be accessible to scientists and investigators via direct request to the authors.
