# Supplementary material for: 1H NMR based metabolic profiling distinguishes the differential impact of capture techniques on wild bighorn sheep
Source: Sci Rep. 2021 May 28;11:11308. doi: 10.1038/s41598-021-90931-y (PMC8163747; doi:10.1038/s41598-021-90931-y)

*Supporting Information (SI):*

Re: "1H NMR Based Metabolic Profiling Distinguishes the Differential Impact of Capture Techniques on Wild Bighorn Sheep" Authors: O’Shea-Stone, G., Lambert, R., Tripet, B., Berardinelli, J., Thomson, J., Copié, V., and Garrott, R

**SI Table S1**. The distribution of blood serum samples collected from 14 wild bighorn sheep herds in Montana and Wyoming between December 2014 and February 2017 that were incorporated into NMR-based metabolic profiling and analyses.

| Herd name | State | No. samples | Sample dates |
| --- | --- | --- | --- |
| Absaroka | Wyoming | 75 | Mar 2015; Mar, Dec 2016;  Jan, Mar 2017 |
| Devil’s Canyon | Wyoming | 25 | Mar 2017 |
| Dubois | Wyoming | 88 | Mar 2015; Mar, Dec 2016;  Mar 2017 |
| Ferris Seminoe | Wyoming | 8 | Feb 2015 |
| Jackson | Wyoming | 66 | Jan, Mar 2015; Mar, Dec 2016;  Jan, Mar 2017 |
| Grand Teton Natl Park | Wyoming | 5 | Jan 2017 |
| Castle Reef | Montana | 47 | Dec 2014; Jan 2015; Dec 2016;  Feb 2017 |
| Fergus | Montana | 88 | Dec 2014; Dec 2016; Feb 2017 |
| Galton | Montana | 14 | Jan 2017 |
| Lost Creek | Montana | 38 | Jan, Dec 2015; Mar 2015; Dec 2016 |
| Yellowstone Natl Park | Montana | 4 | Dec 2014 |
| Perma-Paradise | Montana | 56 | Dec 2014; Dec 2016 |
| Stillwater | Montana | 18 | Dec 2014; Jan, Mar 2015 |
| Taylor-Hilgard | Montana | 63 | Jan 2015; Feb, Dec 2016 |

**SI Figure S1.** Study area map showing the approximate locations of the 14 Montana and Wyoming bighorn sheep populations used in this study. (ESRI 2011. ArcGIS Desktop: Release 10. Redlands, CA: Environmental Systems Research Institute. <https://www.esri.com/en-us/home>)


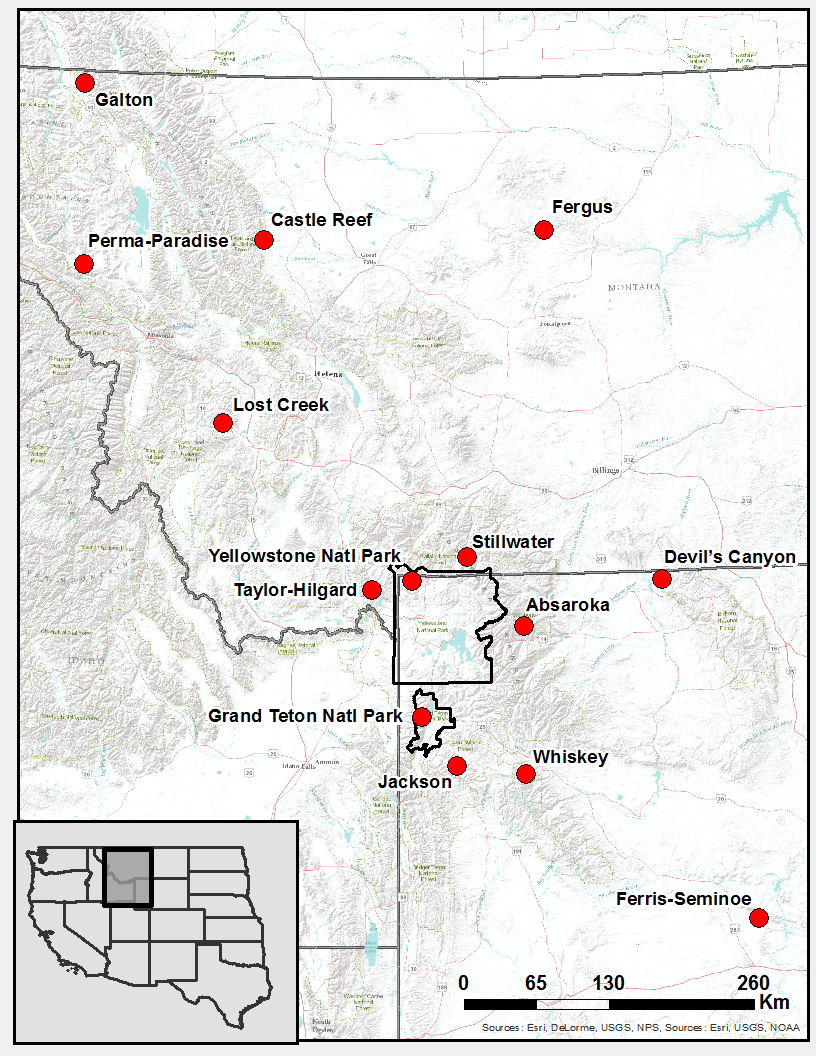


**SI Figure S2.** Classification Error Rate plots and area under ROC curve (AUROC) analyses that were used to validate PLS-DA models for all capture techniques **(A)**, helicopter vs. dart captures **(B)**, dart vs. dropnet captures **(C)** and helicopter vs dropnet captures **(D).** PLS-DA was performed using custom code written in R utilizing the “MixOmics” package. Parameters included using the ‘plsda’ function withing MixOmics, max iterations = 100 and the ‘classic’ algorithm. Five components were included in each model, due to lowest CER in all four analysis at component 5, additionally component 5 is shown in ROC curves corresponding to each analysis.

**SI Fig. S2A.** All capture techniques

CER: All captures


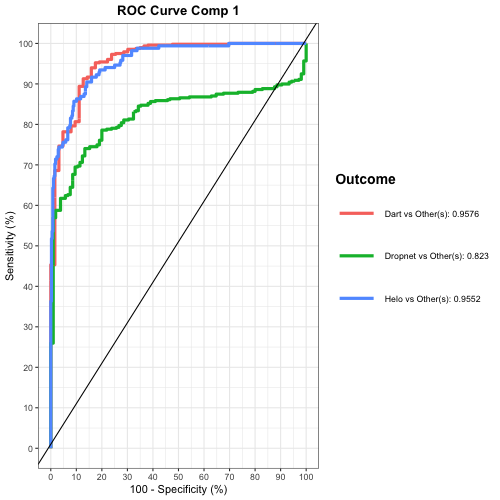


**
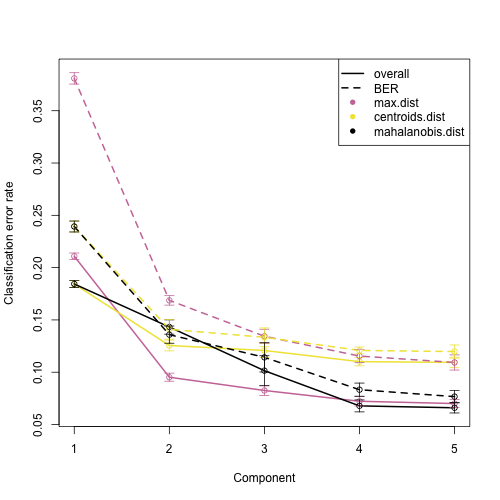
**

**SI Fig. S2B.** Helicopter vs. Dart Capture

CER: Helicopter vs Dart captures


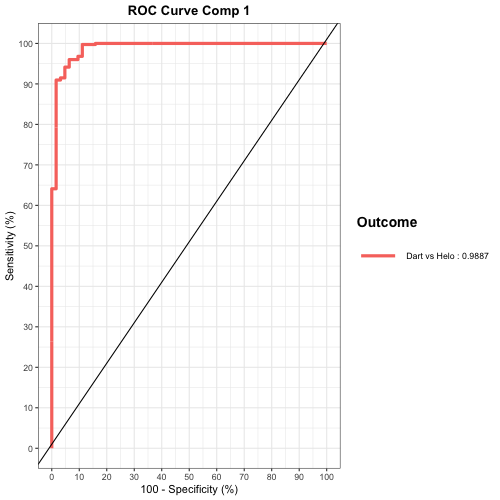


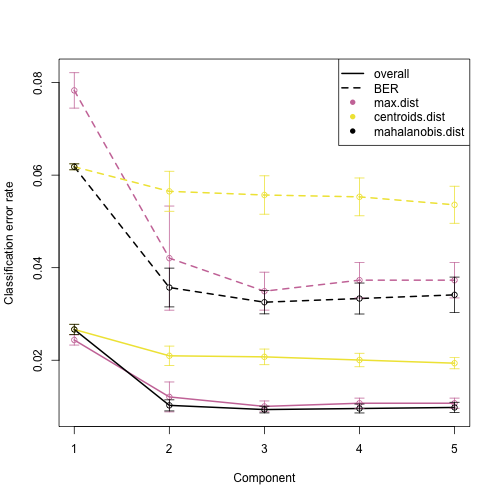


**SI S2C.** Dart vs. Dropnet Capture

CER: Dart vs Dropnet captures


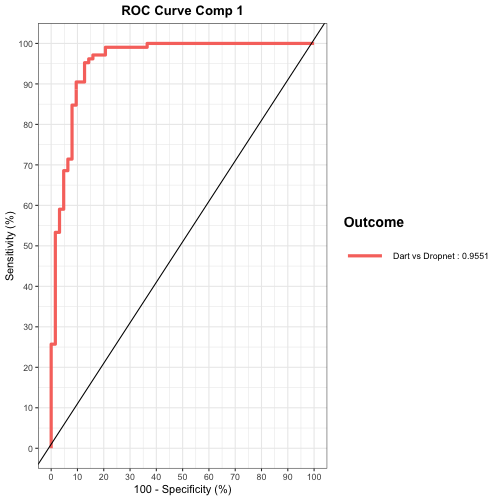


**
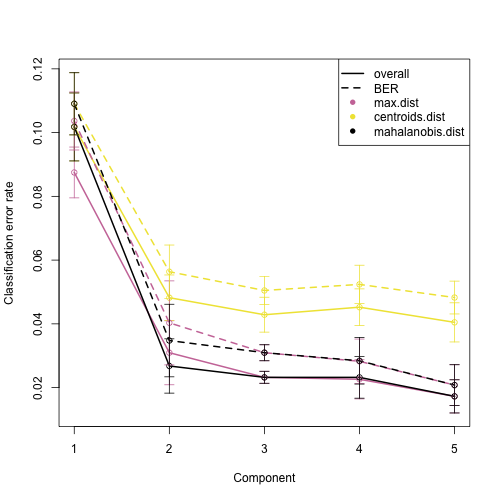
**

**SI Fig. S2D.** Helicopter vs Dropnet Captures

CER: Helicopter vs Dropnet

**
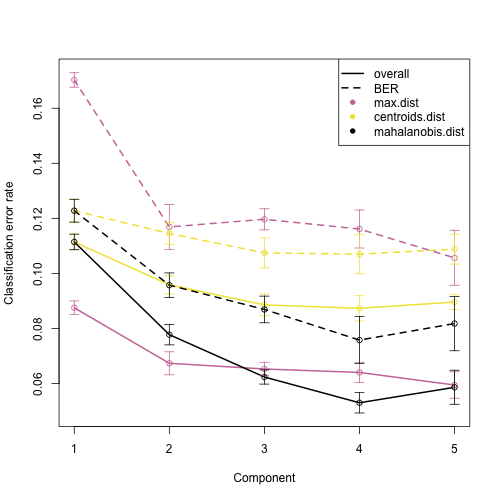
**


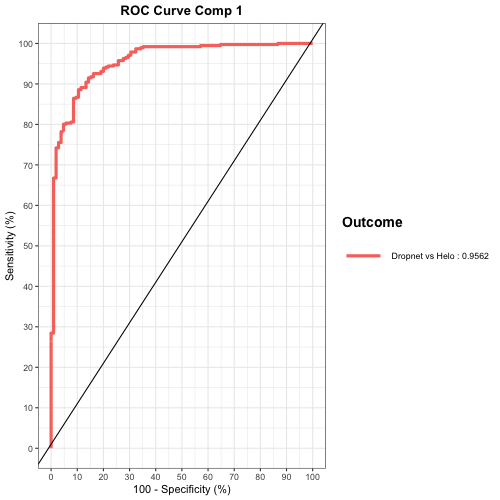


**SI Figure S3.** PLS-DA loading vectors importance values for the model that includes all capture techniques, dart (orange), dropnet (blue) and helicopter captures (green).


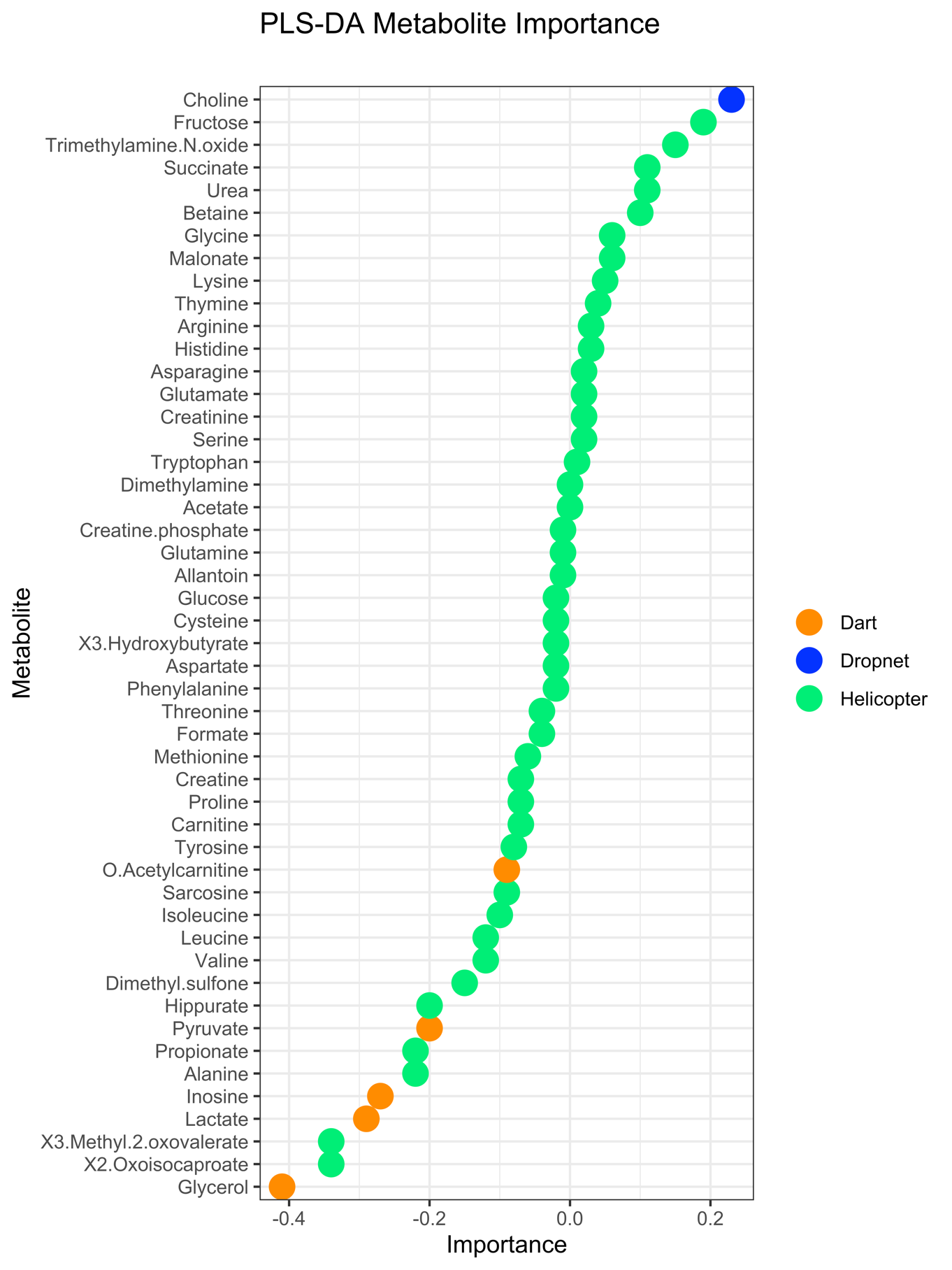


**SI Figure S4.** Volcano plot analysis revealing 18 significant metabolites with fold change (FC) >1.5 and p-value threshold p<0.05 discriminating between the metabolome profiles of dart captured animals (orange) versus the dropnet captured group (blue).

**SI Figure S5.** Volcano plot analysis indicating that only three significant metabolites with fold change (FC) >2.0 and p-value threshold p<0.05 discriminate between the dropnet animal capture (blue) and the helicopter capture technique (green).

**SI Table S2.**  Complete set of metabolites included in all metabolite profile analyses, displaying concentration mean (in μM), standard deviation and statistical significance based on comparison between all capture types. Statistical significance was calculated using non-parametric one-way ANOVA with Tukey’s post-hoc analysis, and a false discovery rate (FDR) adjusted p-value of p < 0.05. Determined from polar metabolite extract 1D **^1^**H NMR spectra.

| **Metabolite** | **Dart Mean ± SD** | **Dropnet Mean ± SD** | **Heli. Mean ± SD** | **p-value** |
| --- | --- | --- | --- | --- |
| 2-Oxoisocaproate | 0.013±0.005 | 0.032±0.009 | 0.024±0.01 | 4.05E-38 |
| 3-Hydroxybutyrate | 0.201±0.064 | 0.228±0.087 | 0.191±0.058 | 1.19E-67 |
| 3-Methyl-2-oxovalerate | 0.016±0.005 | 0.04±0.012 | 0.025±0.011 | 1.33E-55 |
| Acetate | 0.529±0.225 | 0.516±0.194 | 0.424±0.161 | 3.72E-35 |
| Alanine | 0.143±0.052 | 0.266±0.049 | 0.303±0.069 | 3.26E-12 |
| Allantoin | 0.179±0.075 | 0.251±0.087 | 0.294±0.106 | 2.27E-12 |
| Arginine | 0.056±0.046 | 0.051±0.043 | 0.05±0.048 | 4.63E-24 |
| Asparagine | 0.017±0.007 | 0.021±0.01 | 0.021±0.009 | 8.06E-22 |
| Aspartate | 0.012±0.004 | 0.014±0.006 | 0.012±0.006 | 4.32E-43 |
| Betaine | 0.158±0.044 | 0.17±0.04 | 0.193±0.053 | 3.51E-53 |
| Carnitine | 0.051±0.02 | 0.065±0.025 | 0.054±0.018 | 1.84E-50 |
| Choline | 0.01±0.004 | 0.01±0.004 | 0.017±0.006 | 2.42E-12 |
| Creatine | 0.089±0.028 | 0.141±0.059 | 0.148±0.05 | 4.75E-16 |
| Creatine phosphate | 0.013±0.014 | 0.016±0.011 | 0.018±0.013 | 2.10E-10 |
| Creatinine | 0.093±0.032 | 0.094±0.027 | 0.09±0.029 | 7.35E-53 |
| Cysteine | 0.026±0.042 | 0.023±0.009 | 0.022±0.022 | 5.07E-34 |
| Dimethyl sulfone | 0.023±0.021 | 0.044±0.029 | 0.033±0.021 | 9.83E-12 |
| Dimethylamine | 0.016±0.006 | 0.017±0.006 | 0.015±0.005 | 1.13E-59 |
| Formate | 0.06±0.038 | 0.04±0.009 | 0.021±0.012 | 1.77E-120 |
| Fructose | 0.238±0.111 | 0.193±0.101 | 0.254±0.125 | 1.70E-27 |
| Glucose | 5.313±1.555 | 6.02±1.594 | 5.19±1.48 | 1.43E-89 |
| Glutamate | 0.043±0.02 | 0.045±0.019 | 0.045±0.024 | 2.18E-39 |
| Glutamine | 0.071±0.063 | 0.085±0.082 | 0.073±0.078 | 2.40E-10 |
| Glycerol | 0.079±0.134 | 1.052±0.601 | 1.386±0.685 | 1.83E-130 |
| Glycine | 0.172±0.072 | 0.197±0.072 | 0.235±0.068 | 2.69E-16 |
| Hippurate | 0.063±0.033 | 0.123±0.057 | 0.096±0.051 | 2.24E-26 |
| Histidine | 0.017±0.014 | 0.018±0.014 | 0.018±0.013 | 2.94E-11 |
| Inosine | 0.013±0.017 | 0.058±0.038 | 0.092±0.057 | 1.88E-47 |
| Isoleucine | 0.063±0.019 | 0.081±0.021 | 0.064±0.019 | 9.42E-65 |
| Lactate | 3.303±6.041 | 12.896±4.492 | 23.927±5.228 | 2.72E-180 |
| Leucine | 0.078±0.026 | 0.118±0.029 | 0.121±0.032 | 1.03E-29 |
| Lysine | 0.032±0.011 | 0.035±0.01 | 0.035±0.013 | 5.54E-39 |
| Malonate | 0.028±0.018 | 0.025±0.016 | 0.028±0.02 | 1.13E-13 |
| Methionine | 0.013±0.007 | 0.019±0.008 | 0.02±0.009 | 2.52E-12 |
| O-Acetylcarnitine | 0.005±0.004 | 0.013±0.008 | 0.021±0.009 | 5.52E-21 |
| Phenylalanine | 0.025±0.005 | 0.029±0.006 | 0.026±0.008 | 1.61E-69 |
| Proline | 0.062±0.023 | 0.085±0.032 | 0.08±0.02 | 1.28E-51 |
| Propionate | 0.012±0.012 | 0.022±0.015 | 0.014±0.009 | 1.59E-40 |
| Pyruvate | 0.025±0.02 | 0.054±0.014 | 0.08±0.021 | 6.05E-34 |
| Sarcosine | 0.007±0.003 | 0.009±0.003 | 0.007±0.006 | 9.01E-39 |
| Serine | 0.025±0.026 | 0.022±0.028 | 0.022±0.035 | 5.79E-19 |
| Succinate | 0.011±0.006 | 0.01±0.005 | 0.014±0.011 | 6.69E-13 |
| Threonine | 0.034±0.027 | 0.049±0.03 | 0.055±0.027 | 2.10E-05 |
| Thymine | 0.009±0.006 | 0.007±0.003 | 0.007±0.004 | 7.12E-45 |
| Trimethylamine N-oxide | 0.171±0.173 | 0.105±0.07 | 0.141±0.117 | 7.15E-26 |
| Tryptophan | 0.038±0.012 | 0.037±0.008 | 0.029±0.008 | 1.10E-99 |
| Tyrosine | 0.04±0.012 | 0.057±0.011 | 0.06±0.016 | 4.80E-42 |
| Urea | 5.364±5.698 | 3.139±1.212 | 3.197±2.081 | 7.62E-48 |
| Valine | 0.138±0.039 | 0.187±0.036 | 0.153±0.048 | 4.64E-67 |

**SI Table S3.** Variable of Importance (VIP) values for all metabolites included in the PLS-DA model of the three capture techniques, taking into account the first 5 components and listed in descending order of significance based on their ranking in the component 1 dimension.


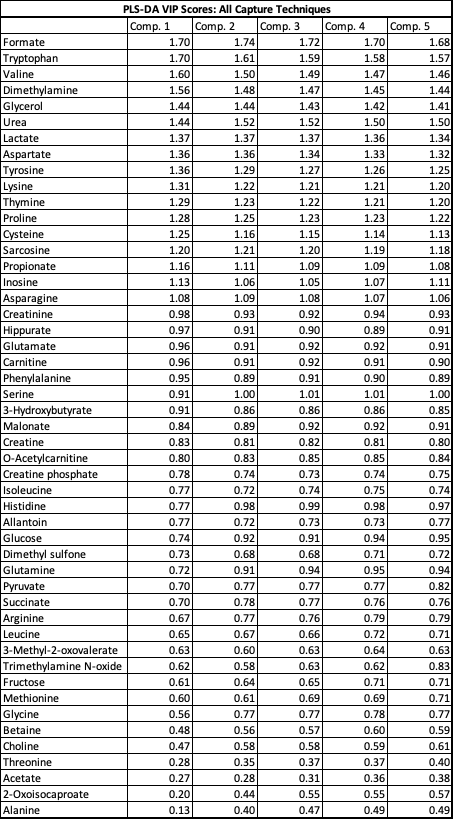


**SI Figure S6.**  PLS-DA loadings plots for all animal capture techniques **(A)**, helicopter vs. dart captures **(B)**, dart vs. dropnet captures **(C)** and helicopter vs dropnet captures **(D).**


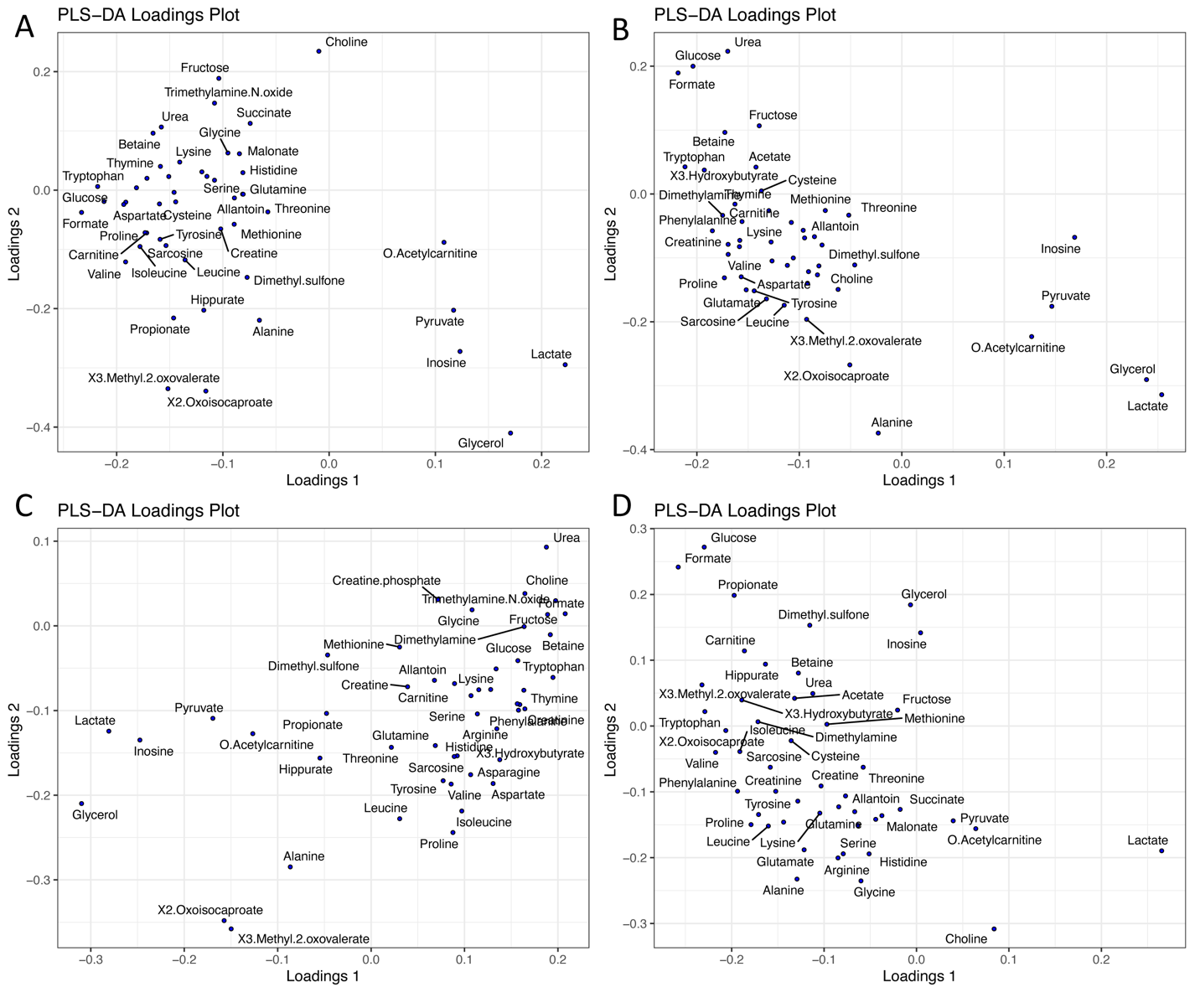

Supplement: Supplementary file 1 — Supplementary Information. [file 41598_2021_90931_MOESM1_ESM.docx]
